# Treatment of peri‐implantitis with diode laser or mucosal flap surgery: A clinical randomized controlled trial

**DOI:** 10.1002/JPER.24-0683

**Published:** 2025-03-24

**Authors:** Sebastian Malmqvist, Talat Qadri, Ronaldo Lira‐Junior, Elisabeth A. Boström, Anders Gustafsson, Georgios N. Belibasakis, Angelika Silbereisen, Gunnar Johannsen, Annsofi Johannsen

**Affiliations:** ^1^ Division of Oral Health and Periodontology, Department of Dental Medicine Karolinska Institutet Huddinge Sweden; ^2^ Kami Dental Enköping Sweden; ^3^ Division of Oral Diagnostics and Surgery, Department of Dental Medicine Karolinska Institutet Huddinge Sweden; ^4^ Department of Orofacial Medicine Public Dental Health Services Stockholm Stockholm Sweden

**Keywords:** laser therapy, peri‐implantitis, peri‐implantitis surgery, randomized controlled trial

## Abstract

**Background:**

Peri‐implantitis poses a major challenge as contemporary nonsurgical treatments show dissatisfactory results and mucosal flap surgery is often needed. Diode lasers can remove granulation tissue and submucosal bacteria, and if it achieves similar clinical results, then it could be used as a less invasive first approach. The aim was to compare the healing of peri‐implantitis lesions 6 months after treatment with either diode laser or conventional mucosal flap surgery.

**Methods:**

In this clinical randomized controlled trial, 26 patients with peri‐implantitis were treated with either 970 nm diode laser (1.2 W, continuous wave) or mucosal flap surgery. Clinical variables, marginal bone level (MBL), inflammatory biomarkers, and submucosal pathogens were examined at baseline and 6 months after treatment. Patients graded their pain and discomfort at baseline, directly after treatment, after first week of healing (FWH), and after 6 months. The continued need of care was also noted after 6 months.

**Results:**

Equivalence was not shown between treatments in probing pocket depth (PPD) and MBL. Only plaque index (PI) showed significant changes between groups (*p *= 0.023). There was a significant difference between surgery (−1.81 ± 0.94 mm) and laser (−0.83 ± 0.40 mm), among those that improved their PPD (*p *= 0.016). Pain and discomfort were rated low in both groups. Negligible differences were found regarding immunological markers and submucosal bacteria.

**Conclusions:**

The proportion of patients with improved clinical outcomes was similar between the 2 treatment groups, albeit surgical treatment yielded greater pocket reduction. No differences were found in immunological or microbial outcomes.

**Plain Language Summary:**

Replacing missing teeth with dental implants has become a popular treatment as it is a fixed solution compared to removable dentures. In some cases, the tissue surrounding the dental implant becomes inflected, which can lead to a breakdown of the bone in which the implant is attached; this disease is called peri‐implantitis. Treating peri‐implantitis has proven to be difficult. Few studies have previously investigated the treatment of peri‐implantitis with infra‐red laser. The goal was to compare if the healing of the peri‐implantitis disease after treatment was comparable between infrared diode laser and conventional surgery, with emphasis on the patients’ experiences. The healing was evaluated with clinical examination and assessment of surrounding bone on X‐rays, as well as immune response and bacterial samples. We could not statistically confirm that the two treatments were equal in healing outcomes, but they had similar numbers of successful and unsuccessful healing patterns after six months. The surgery had some clinical advantages in the severe cases of peri‐implantitis and the laser resulted in less discomfort during the first week of healing.

## 1 INTRODUCTION

Maintaining a healthy peri‐implant condition is a major challenge in dentistry, given that dental implants have become a common therapy for replacing missing teeth, with estimated yearly sales reaching 12–18 million worldwide.^1^ Controlling the biofilm with regular supportive peri‐implant care and the patient's own oral hygiene efforts at home is essential in preventing peri‐implant diseases, which pose a risk for the long‐term survival of the implant.^2^, ^3^ The prevalence of peri‐implantitis is estimated to be 19.83% on a patient‐level and 9.25% on an implant‐level, with worldwide new yearly cases estimated to be 1.1–1.7 million.^1^, ^4^

Deploying nonsurgical treatments, such as mechanical debridement, of peri‐implant mucositis has shown positive results, while yielding irregular outcomes in peri‐implantitis.^5^ The standard treatment of an established peri‐implant lesion is mucosal flap surgery, to gain access to the implant surface for mechanical decontamination and to remove inflamed tissue,^6^ in combination with supportive maintenance therapy.^7^, ^8^ Satisfactory results have been shown up to 3 years post‐surgery,^9^ although at a follow‐up after 5 years ∼20% of implants affected with peri‐implantitis needed to be, or were removed.^10^ A variety of different adjunctive treatment alternatives such as submucosal air polishing, local and systemic antibiotics, various antiseptic chemical agents, and lasers have been tested.^11^, ^12^

The Er:YAG laser, suitable for hard tissue removal, that is, mineralized deposits, is the most commonly tested laser in periodontology, but has shown contradictory results as an adjunctive treatment to both surgical and nonsurgical treatment of peri‐implantitis.^13^, ^14^, ^15^ Diode lasers have shown promising results in decontaminating implant surfaces without damaging them,^16^, ^17^, ^18^ but care must be taken to not cause heat‐related damage to the surrounding bone.^19^

The effect of diode lasers occurs through direct disinfection of bacteria as well as removal of the surrounding infected soft tissue.^20^ There is, however, a lack of studies on diode lasers,^21^, ^22^, ^23^ and to the best of our knowledge no clinical randomized controlled trials (RCTs), with at least 6 months follow‐up, have been published on the 970 nm laser used to remove the submucosal biofilm, diseased epithelium, and granulation tissue around dental implants. If diode lasers can achieve similar clinical results as mucosal flap surgery, then it could be used as a less invasive first approach, especially if patients experience the treatment as less painful or discomfortable.

An often‐overlooked aspect of evaluating treatments is the patient‐reported outcome measures (PROM), such as pain and satisfaction. In a thorough review of the published studies on treating peri‐implantitis up to April of 2021, as few as 6% of the studies included PROM.^24^ This highlights the importance of including PROM in research that assesses treatment of peri‐implantitis.^25^

Thus, the aim of this study was to compare the healing of peri‐implantitis lesions 6 months after treatment with either diode laser or conventional mucosal flap surgery, primarily looking at equivalence for mean change in probing pocket depth (PPD) and marginal bone level (MBL), but also differences in other clinical variables, patient‐reported outcomes, and inflammatory and microbial responses.

## 2 MATERIALS AND METHODS

### 2.1 Study design

This study was designed as a parallel group RCT comparing laser treatment to conventional mucosal flap surgery after 6 months, with main outcomes of equivalence and secondary outcomes explored for superiority. The study was conducted and reported in accordance with the Consolidated Standards of Reporting Trials (CONSORT) guidelines,^26^ the CONSORT extension of noninferiority and equivalence trials,^27^ and the Declaration of Helsinki.^28^ The study was registered prospectively at ClinicalTrials.gov (NCT04249024). Ethical approval was obtained from the regional ethics committee in Stockholm (Dnr. 2015/822‐31/2). Written and oral informed consent was obtained from the participants before enrollment.

### 2.2 Study population and recruitment

Participants were recruited from patients referred for treatment of peri‐implantitis at a specialist clinic (Danakliniken, Specialist Dental Care, Stockholm, Sweden) between October 2019 and November 2021. All included patients had clinical and radiographic signs of peri‐implantitis surrounding at least 1 dental implant and fulfilled the following eligibility criteria.

#### 2.2.1 Inclusion criteria

- At least 1 dental implant with peri‐implantitis defined as:
  ∘ Presence of PPD ≥ 6 mm.
  ∘ Bleeding/suppuration on probing (BOP/SOP).
  ∘ At least 2 mm loss of bone on radiographs, after initial osseointegration or when the history of the implant was not available from assumed initial bone level based on implant design.^29^
- ≥ 18 years old.

#### 2.2.2 Exclusion criteria

- Antibiotic treatment 6 months prior to baseline.
- Peri‐implant treatment of the affected dental implants 6 months prior to baseline.
- Myocardial infarction 6 months prior to baseline.
- Previous radiation treatment in the affected jaw area.
- Previous intravenous bisphosphonate treatment.

### 2.3 Sample size, randomization, and allocation

An a priori sample size calculation was made testing for equivalence of PPD between treatments based on the average improvement shown in a meta‐analysis by Chen et al.^30^ of 2.38 mm (± 0.53 standard deviation, SD). Equivalence limit of 1.0 for the confidence interval, power (1‐β) 80%, and level of significance α = 0.05, at least 20 participants in each group were needed and with a margin for dropouts a total of 50 participants were planned to be included. However, due to the coronavirus disease 2019 (COVID‐19) pandemic enrolment had to be adapted to all eligible patients between October 2019 and November 2021.

**FIGURE 1:**
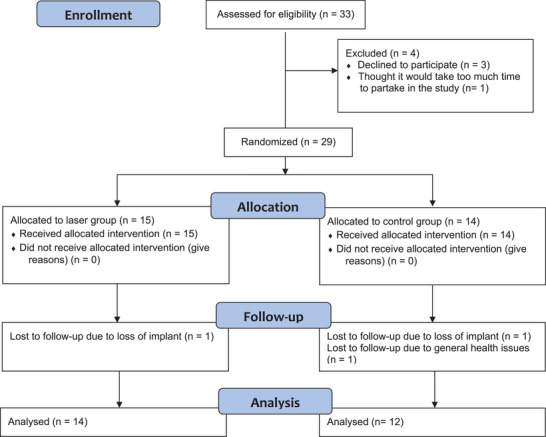
CONSORT 2010 flow diagram

The patients were screened for eligibility by a periodontist (G.J.) and participants were, after baseline examination (by S.M.), randomized to either treatment group by means of block randomization stratified for baseline average PPD around most severely affected dental implant. A premade list of allocations was used sequentially with random order of block sizes of 4 or 6 for each stratum, which were < 4.5 mm, 4.5–6.5 mm, and > 6.5 mm. 33 potential participants were invited and 26 completed the study (Figure 1).

### 2.4 Intervention

All participants were given individual instructions in oral hygiene (by S.M.) after baseline examination (before allocation), at the early follow‐up (7–12 days), and the 6 months follow‐up. The implant supported prosthesis was not removed for the treatments or examinations. Both treatments were carried out under local anesthesia, and the patients were instructed to rinse daily with 0.2% chlorhexidine solution until their first follow‐up visit.

The experimental group received (by S.M.) flapless mechanical debridement with steel curettes, in cases with calculus, and then treatment with a 970 nm diode laser*. The laser device was used in continuous wave mode, 1.2 W power, 320 µm fiber diameter†, and manual irrigations with sterile saline solution (0.9% NaCl). Settings were based on a previous unpublished pilot study and safety aspects from a previous study.^19^ During activations, the laser fiber was moved back and forth while gradually moving from the marginal peri‐implant mucosa to the bottom of the pocket. Treatment was carried out until the whole area of the peri‐implant pocket had been irradiated to potentially remove all of the diseased epithelium and granulation tissue.

The control group was treated (by G.J.) with conventional mucosal flap surgery. The surgical procedure involved an elevation of a mucoperiosteal flap after a reversed bevel incision, after which all granulation tissue were removed, the exposed threads of the implants were brushed with a titanium brush‡, and concluded by suturing of the mucosal flap. The patients returned after 7–12 days for removal of the sutures.

### 2.5 Outcomes

Outcomes were measured at baseline and 6 months after treatment, with additional measurement of PROM directly and 7–12 days after treatment to grade the first week of healing (FWH). Clinical variables, PROM, and microbial analyses were not blinded for the assessor, whereas measurements of bone level on radiographs and immunological markers were blinded for the assessors. The patients were not blinded as the treatment methods had obvious differences. Primary outcomes were mean change in PPD and MBL from baseline to follow‐up, and examined for equivalence, while other variables were treated as secondary outcomes.

#### 2.5.1 Clinical and radiographic outcomes

Clinical measurements were recorded at 4 sites per implant (mesial, buccal, distal, and lingual/palatal) with a millimeter‐graded periodontal probe§ and analyzed primarily on implant‐level. Four patients (16 sites) were remeasured for PPD 2–4 weeks after baseline to determine an intraclass correlation of the examiner, which was 0.989 (95% CI: 0.975 – 0.995). Radiographs were taken (by S.M.) and MBL assessed by consensus discussion between 2 authors (A.J. and T.Q.).

- Plaque index (PI) was registered as presence/absence of visual plaque.
- BOP and SOP were evaluated as presence/absence of bleeding and suppuration respectively, after probing and within 30 s.
- PPD was assessed from the mucosal/gingival margin to the bottom of the peri‐implant/periodontal pocket.
- MBL was measured on periapical radiographs in Fiji Image J 1.53c^31^ from the point of estimated original bone level to the current MBL along the implant. The implant's length or distance between threads were used to calibrate the distance in mm.

#### 2.5.2 Patient‐reported outcomes

Participants graded their pain, discomfort, and satisfaction on a 100 mm visual analogue scale (VAS). The questions were explained by either S.M. or G.J. beforehand and then the participants were allowed to answer them in their own time but with the option to ask for clarifications.

#### 2.5.3 Immunological outcomes

Sampling of stimulated saliva was performed at the start of each examination. Prior to the visit, the participants were requested not to smoke, brush their teeth, eat, or drink, except water, 1 h before the visit. Stimulated saliva was collected for 5 min, during which the participants chewed on a piece of paraffin, and then it was stored on ice until transported to the laboratory. The saliva was then centrifuged at 1500 *g* for 10 min at 4°C, supernatants were collected, aliquoted, and placed in a −80°C freezer until analysis.

Peri‐implant crevicular fluid (PICF) was collected from both the mesial and distal sides of the implant with deepest bone defect, assessed by periapical radiographs. A paper strip‖ was inserted in the mesial peri‐implant pocket for 30 s, without causing bleeding, and then placed in a micro moisture meter¶ The procedure was repeated for the distal site and both strips placed in the same 2 mL tube, with 200 µL phosphate buffered saline (PBS), and stored on ice until frozen at −80°C. The linear regression based on a standard curve of known volumes was used to calculate the volume, as suggested by Ciantar and Caruana.^32^

For both stimulated saliva and PICF, concentrations of calprotectin (S100A8/A9), interleukin‐1 beta (IL‐1β), and matrix‐metalloproteinase‐8 (MMP‐8) were determined by enzyme‐linked immunosorbent assay kits, according to the manufacturer's instructions# Readings were made using a microplate spectrophotometer** at 450 nm with wavelength correction set to 540 nm.

#### 2.5.4 Microbiological outcomes

The submucosal biofilm samples collected by medium sized sterile paper points†† from the same implant sampled for biomarkers, and the mesial and distal sites were pooled in a 2 mL tube, to then be frozen at −80°C until analysis. 100 µL PBS was added to each tube and the samples incubated overnight at 600 rpm (4°C). Thereafter, samples were vortexed for 1 min followed by 1 min of sonication. Paper points were then removed, and remaining samples were subjected to total genomic DNA extraction using a bacterial genomic DNA kit‡‡ The extracted DNA was frozen at −20°C prior to quantitative real‐time polymerase chain reaction (qPCR) to determine the log_10_ counts of *Porphyromonas gingivalis*, *Fusobacterium nucleatum*, *Tannerella forsythia*, and *Treponema denticola*, as described earlier.^33^ For the qPCR 20 µL reaction volume was used (2.0 µL samples or controls, 6.0 µL nuclease‐free water, 1.0 µL each of forward and reverse primer, and 10.0 µL qPCR master mix§§ Reference genomic DNA and species‐specific primers are provided in Table S1 in online *Journal of Periodontology*.

### 2.6 Statistical analysis

Shapiro–Wilks test and visual examination (box plots) were used to test for normality of the data. Statistical analyses were performed per‐protocol with a commercially available statistical software‖‖ and an open‐source software JASP (versions 0.16 and 0.17.2.1; JASP Team^34^). The primary outcomes were assessed for equality between the treatment groups with a predefined zone of equivalence of ± 1.0 and tested using the two one‐sided test (TOST).^35^, ^36^ Other analyses performed were to test for differences between the groups by using the parametric Student's *t*‐test or nonparametric Mann–Whitney *U*‐test or Wilcoxon signed‐rank test. For nominal data, the chi squared test was applied. Level of significance was set at α = 0.05.

## 3 RESULTS

### 3.1 Participants’ characteristics

Out of the 33 patients screened for inclusion in the study, 26 participants (29 affected implants), 14 in the laser group and 12 in the surgery group, completed the study (Figure 1). The mean follow‐up time was 219.3 ± 22.3 days. Age, sex, jaw placement of implant, and implant brand were similarly distributed between the groups (Table 1). There were more current smokers in the surgery group, compared to the laser group, and more former smokers in the laser group, although the difference in distribution was not significant. All included implants had a modified surface, and their prosthesis were screw‐retained. The average irradiation time and total energy for each treated dental implant in the laser group were 314 ± 142 s and 376 ± 170 J, respectively.

**TABLE 1:** Background information about the participants in the study.

Characteristic	Laser group	Surgery group	*p*‐value	Total
Patients, *n*	14	12		26
Dental implants, *n* (mean ± SD)	62 (4.4 ± 2.2)	41 (3.4 ± 2.4)	0.273	103 (4.0 ± 2.3)
Peri‐implantitis implants, *n*	17	12	0.088^a^	29
Teeth, mean ± SD	14.6 ± 8.7	20.8 ± 6.1	0.050	17.4 ± 8.1
Age, mean ± SD	67.6 ± 14.5	63.8 ± 12.6	0.198^b^	65.8 ± 13.5
Sex, *n* (%)				
Female	11 (76.9%)	9 (75.0%)	0.829^a^	20 (76.9%)
Male	3 (21.4%)	3 (25.0%)		6 (23.1%)
Tobacco smoking, *n* (%)				
Smokers	2 (14.3%)	5 (41.7%)	0.089^a^	7 (26.9%)
Former smokers	7 (50.0%)	2 (16.7%)		9 (34.6%)
Non‐smokers	5 (35.7%)	5 (41.7%)		10 (38.5%)
Implant position, *n* (%)				
Maxilla	13 (76.5%)	8 (66.7%)	0.544^a^	21 (72.4%)
Mandible	4 (23.5%)	4 (33.3%)		8 (27.6%)
Implant brand, *n* (%)				
Company A^c^	10 (58.8%)	8 (66.7%)	0.177^a^	18 (62.1%)
Company B^d^	3 (17.6%)	2 (16.7%)		5 (17.2%)
Company C^e^	0 (0%)	2 (16.7%)		2 (6.9%)
Other	4 (23.5%)	0 (0%)		4 (13.8%)

*Note*: Independent *t*‐test to compare means.

Abbreviations: *n*, number; SD, standard deviation.

^a^ Chi^2^, to compare distributions

^b^ Mann‐Whitney *U*‐test, non‐normally distributed means

Company behind each implant brand:

^c^ Straumann

^d^ Nobel Biocare

^e^ Astra Tech

### 3.2 Clinical and radiographic outcomes

This study failed to show equivalence between the treatment methods (Table 2). For PPD, the TOST showed that the upper bound of the zone of equivalence was breached, while for MBL the lower bound was just barely crossed (Table 2).

**TABLE 2:** Test for equivalence between treatments regarding PPD and MBL, with the zone of equivalence being (‐1, 1) for both variables.

	Laser		Surgery		Equivalence, CI 100% × (1‐2α)			
Variable	Mean 6 m‐B	SD	Mean 6 m‐B	SD	CI 90% lower	*p*‐Value lower	CI 90% upper	*p*‐Value upper
PPD [mm]	−0.22	±0.91	−0.90	±1.84	−0.29	0.003	1.62	0.280
MBL [mm]	−0.06	±1.02	0.32	±1.18	−1.11	0.081	0.36	0.002

*Note*: p‐Values calculated with Two One‐Sided Test.

Abbreviations: 6 m, 6 months; B, baseline; CI, confidence interval;MBL, marginal bone level; PPD, probing pocket depth; SD, standard deviation.

Among those patients whose PPD improved (*n* = 9 surgery and *n* = 8 laser), there was a significant difference (*p *= 0.016) between the groups in favor of the surgical treatment with a mean decrease of 1.81 ± 0.94 mm, compared to the laser group with a mean decrease of 0.83 ± 0.40 mm. However, there were no significant (*p *= 0.836) difference between groups among patients who improved in MBL (*n* = 6 surgery and *n* = 7 laser), 0.62 ± 0.61 mm and 0.80 ± 0.64 mm for respective groups.

No clinical or radiographic variable differed significantly at baseline between the groups, neither on full‐mouth level (see Table S2 in online *Journal of Periodontology*) nor on peri‐implantitis‐affected implant level (Table 3). On implant level, both groups showed nonsignificant decreases of BOP, SOP, and PPD, compared to baseline. MBL was essentially unchanged in both groups from baseline to follow‐up. Only the change in PI was significantly different between the groups (*p *= 0.023), in favor of the laser group. No other variable at any timepoint or mean change were significantly different.

**TABLE 3:** Clinical and radiographic outcomes of the affected dental implants.

Variable	Baseline (B)			6 Months (6 m)			Change (6 m—B)		
	Laser	Surgery	*p*1	Laser	Surgery	*p*1	Laser	Surgery	*p*1
PI [%] *p*2	43.8 ± 45.7 (19.83, 67.67)	37.5 ± 34.5 (17.96, 57.04)	0.790^a^	33.0 ± 35.9 (14.23, 51.84)	60.4 ± 45.8 (34.50, 86.33)	0.183^a^	−10.7 ± 31.7 (−27.33, 5.90) *p* = 0.228	22.9 ± 39.1 (0.79, 45.04) *p* = 0.067	**0.023**
BOP [%] *p*2	97.3 ± 7.2 (93.5, 101.1)	97.9 ± 7.2 (93.8, 102.0)	0.711^a^	92.0 ± 16.7 (83.2, 100.7)	83.3 ± 28.9 (67.0, 99.7)	0.489^a^	−5.4 ± 15.3 (−13.4, 2.7) *p* = 0.269^b^	−14.6 ± 29.1 (−31.1, 1.9) *p* = 0.174^b^	0.510^a^
SOP [%] *p*2	46.4 ± 37.8 (26.63, 66.23)	43.8 ± 41.5 (20.29, 67.21)	0.914^a^	26.8 ± 36.0 (7.94, 45.63)	41.7 ± 45.6 (15.84, 67.49)	0.431^a^	−19.6 ± 41.8 (−41.5, 2.3) *p* = 0.102	−2.1 ± 29.1 (−18.56, 14.39) *p* = 1.000^b^	0.177^a^
PPD [mm] *p*2	6.30 ± 1.35 (5.59, 7.00)	6.88 ± 1.11 (6.25, 7.50)	0.247	6.07 ± 1.71 (5.18, 6.97)	5.98 ± 2.00 (4.85, 7.11)	0.836^a^	−0.22 ± 0.91 (−0.70, 0.25) *p* = 0.372	−0.90 ± 1.84 (−1.94, 0.14) *p* = 0.120	0.238
MBL mean [mm] *p*2	5.08 ± 2.01 (4.02, 6.13)	5.79 ± 1.91 (4.71, 6.87)	0.403^a^	5.02 ± 2.59 (3.67, 6.38)	6.11 ± 2.82 (4.52, 7.71)	0.314	−0.06 ± 1.02 (−0.59, 0.48) *p* = 0.844	0.32 ± 1.18 (−0.35, 0.99) *p* = 0.365	0.392
MBL mesial [mm] *p*2	4.89 ± 2.27 (3.70, 6.08)	5.63 ± 1.87 (4.58, 6.69)	0.297^a^	4.94 ± 2.84 (3.46, 6.43)	6.13 ± 2.94 (4.39, 7.86)	0.319	0.06 ± 1.05 (−0.50, 0.61) *p* = 0.848	0.45 ± 1.38 (−0.36, 1.27) *p* = 0.981	0.422
MBL distal [mm] *p*2	5.26 ± 1.86 (4.29, 6.24)	5.95 ± 2.11 (4.76, 7.15)	0.384	5.10 ± 2.42 (3.83, 6.37)	6.20 ± 2.90 (4.56, 7.84)	0.301	−0.17 ± 1.13 (−0.76, 0.43) *p* = 0.595	0.25 ± 1.23 (−0.45, 0.95) *p* = 0.500	0.382

*Note*: Values expressed as means ± standard deviation (95% CI). Independent *t*‐test *p*1 between groups and paired *t*‐test *p*2 baseline to 6 months.

Abbreviations: BOP, bleeding on probing; CI, confidence interval; MBL, marginal bone level; PI, plaque index; PPD, probing pocket depth; SOP, suppuration on probing.

Significant *p*‐values in bold.

^a^ Mann‐Whitney *U*‐test, non‐normally distributed means between groups.

^b^ Wilcoxon signed‐rank test, non‐normally distributed means for change within groups.

### 3.3 Patient‐reported outcomes

Overall, the levels of pain and discomfort were rated low in both groups (Table 4). Neither group reported significantly higher levels of pain or discomfort directly after treatment and only in the surgery group did the patients report a significantly higher pain level during the FWH (*p *= 0.026). The participants who received the surgical treatment reported significantly (*p *= 0.010) higher levels of discomfort (VAS 24.9 ± 12.8 mm) than the ones who received laser treatment (VAS 12.0 ± 12.6 mm), during the FWH. Satisfaction remained at a high rating for both groups throughout the study period, although the surgery group reported significantly lower satisfaction at FWH (*p *= 0.022) and 6 months (*p *= 0.047) compared to directly after.

**TABLE 4:** Patient‐reported outcomes of pain and discomfort at the different timepoints.

Variable and group	Baseline	Direct	*p*2	Memory	*p*3	FWH	*p*3	6 Months	*p*3
Pain									
Laser	8.6 ± 16.9 (−0.2, 17.5)	10.4 ± 12.2 (4.0, 16.8)	0.147^a^	12.0 ± 12.7 (5.4, 18.7)	0.547	15.7 ± 17.7 (6.4, 24.9)	0.287	1.5 ± 2.0 (0.4, 2.6)	**0.004** ^a^
Surgery	4.8 ± 7.0 (0.8, 8.7)	6.0 ± 12.7 (−1.2, 13.2)	0.843^a^	13.0 ± 12.9 (5.7, 20.3)	0.065^a^	28.5 ± 25.7 (14.0, 43.1)	**0.026**	3.1 ± 6.6 (−0.6, 6.9)	0.342^a^
*p*1	0.876	0.148		0.643		0.068		0.543	
Discomfort									
Laser	13.5 ± 17.3 (4.4, 22.5)	8.9 ± 9.5 (3.9, 13.9)	0.625^a^	11.4 ± 10.8 (5.8, 17.1)	0.131	12.0 ± 12.6 (5.4, 18.6)	0.468	9.2 ± 11.8 (3.0, 15.4)	0.910
Surgery	15.5 ± 16.8 (6.0, 25.0)	13.7 ± 21.0 (1.8, 25.6)	0.811	12.5 ± 15.7 (3.6, 21.4)	0.248^a^	24.9 ± 12.8 (17.7, 32.1)	0.154	10.7 ± 16.9 (1.1, 20.3)	0.572
*p*1	0.777	0.571		0.959		**0.010**		1.000	
Satisfaction									
Laser		92.7 ± 7.9 (88.6, 96.9)				94.3 ± 7.4 (90.4, 98.2)	0.505	90.9 ± 13.1 (84.1, 97.8)	0.959^a^
Surgery		96.6 ± 5.2 (93.7, 99.6)				87.7 ± 17.4 (77.823, 97.510)	**0.022** ^a^	92.1 ± 8.5 (87.3, 96.9)	**0.047** ^a^
*p*1		0.234				0.254		0.918	

*Note*: Values expressed as mean VAS rating ± standard deviation. Mann‐Whitney *U*‐test: *p*1 comparison between groups. Paired *t*‐test: *p*2 baseline to directly after and *p*3 directly after treatment to respective timepoint.

Abbreviation: FWH, first week of healing.

Significant *p*‐values in bold.

^a^ Wilcoxon signed‐rank test within the groups.

### 3.4 Immunological outcomes

There were no significant changes in the levels of included biomarkers in stimulated saliva or PICF, micro moisture value, or PICF volume between the groups or at follow‐up (see Table S3 and S4 in online *Journal of Periodontology*). S100A8/A9 levels showed a nonsignificant decrease between baseline and follow‐up in the laser group, while an increase was shown in the surgery group (*p *= 0.085). The same marker, in PICF, increased significantly at follow‐up in the laser group (*p *= 0.035) and showed a numerically similar change in the surgery group, although nonsignificant.

### 3.5 Microbiological outcomes

There were generally no significant changes in the level of those species between or within groups over the study period (See Table S5 in online *Journal of Periodontology*). There was a tendency (*p *= 0.052) for higher *P. gingivalis* counts in the laser group compared to the surgery group at baseline, which was reflected by a significant difference between the groups at 6 months (*p *= 0.030) as well.

### 3.6 Post study assessment of future need

There was a nearly significant difference between the groups in distribution of the need for poststudy care (*p *= 0.050), with more participants directly resuming ordinary maintenance care in the surgery group than in the laser group, who in greater extent needed an extra follow‐up visit (Table 5).

**TABLE 5:** Assessment of patients’ future need after the examination at 6 months.

Next appointment	Laser	Surgery
Resume ordinary maintenance care	3 (21.4%)	7 (58.3%)
Extra revisit specialist	7 (50.0%)	1 (8.3%)
Re‐treatment at specialist	4 (28.6%)	4 (33.3%)
χ^2^ *p*‐value	0.050	

## 4 DISCUSSION

The present study could neither establish equivalence between the conventional surgery and diode laser treatment of peri‐implantitis, regarding mean changes in PPD and MBL, nor demonstrate significant differences in clinical, radiographical, immunological, and microbiological outcomes. The surgery had a significant advantage over the diode laser in reduction of PPD, when examining a subgroup of those who improved in that regard.

To the best of our knowledge, this is the first parallel arm RCT evaluating the 970 nm diode laser to remove granulation tissue and submucosal biofilm in treating peri‐implantitis. Only 2 other parallel‐arm RCT:s^37^, ^38^ and 1 split‐mouth RCT^39^ have been published on using other diode lasers as an adjunctive to nonsurgical mechanical debridement. Neither of the studies showed any significant results favoring the adjunctive use of diode lasers (940 nm and 810 nm), with different treatment protocol and laser settings in all 3 studies. Adequate comparisons between those studies and the present one could be further hindered by different use of the diode lasers, low powered solely antimicrobial approach as opposed to our more extensive antimicrobial treatment with granulation tissue removal, as well as different control interventions. Of the 3 previously mentioned studies, only Roccuzzo et al.^37^ included patients with more than just mild peri‐implantitis, with inclusion criteria of MBL loss of ≥2 mm, the same as the present study. Both Alpaslan Yayli et al.^38^ and Arısan et al.^39^ excluded patients with MBL loss exceeding 3 mm. Despite similar inclusion criteria, Roccuzzo et al.^37^ had on average less severe peri‐implantitis patients in their study with baseline averages of PPD 5.29–5.40 mm and MBL 2.04–2.09 mm compared to the present study's PPD 6.30–6.88 mm and MBL 5.08–5.79 mm. The complexity and severity of the disease or the included patient cohort composition could potentially explain the difference in clinical outcomes between the studies, where they managed a greater reduction of PPD and SOP. MBL did not change significantly in either study.

One RCT has examined a 980 nm diode laser to disinfect the implant surface during mucosal flap surgery.^40^ They could not show a significant advantage for the adjunctive use of diode laser during surgery. Having access to the implant surface during flap surgery seems to facilitate adequate decontamination by mechanical means. Our laser protocol aims to decontaminate the implant but also some of the infected soft tissue surrounding the implant. It remains to be seen, in a larger scope, if this approach could yield equivalent results as mucosal flap surgery.

Tenore et al.^41^ have in their RCT on peri‐implant mucositis and initial peri‐implantitis shown promising results on PPD and BOP at 3 months, using a 980 nm diode laser as “soft tissue laser curettage”. Although they had other laser settings and protocol than our study, they showed some advantages for the more extensive soft tissue focused laser treatment. It is, however, unclear how many initial peri‐implantitis cases that were included in their study as they only reported PPD and BOP.

Our study and the one by Roccuzzo et al.^37^ are in agreement concerning nonsignificant change, for both groups, in concentration of IL‐1β in PICF. They found significantly lower levels of MMP‐8 in the laser group at 6 months compared to baseline, while we could not demonstrate a change in that marker.

Neither we nor Roccuzzo et al.^37^ showed significant changes in submucosal biofilm counts of *P. gingivalis*, *T. forsythia*, and *T. denticola*. Roccuzzo et al.^31^ found significantly lower counts of *F. nucleatum* at 6 months compared to baseline. Either the treatments, on average, did not achieve an ecological shift in the submucosal biofilm, or it happened and then regressed over the 6‐month follow‐up period, as the peri‐implant pocket gets recolonized by pathogens after therapy.^42^

In general, both treatments resulted in low ratings of pain and discomfort. In the surgery group, discomfort was rated higher during FWH, probably due to the sutures, which has previously been noted as a potential negative experience of the treatment.^43^

A longer follow‐up period would have revealed the need for further surgery or removal of implants. Carcuac et al.^10^ has shown a peri‐implantitis recurrence in 43.8% (57 out of 130) of implants, out of which 27 implant needed removal within 5 years of surgical treatment. In our study, four patients in each group had an implant in need of re‐treatment, which is in line with findings by Roccuzzo et al.^37^ Since re‐treatment with laser is easier to perform and more convenient for the patients this would be preferable if the long‐term results are proven equivalent to surgery. Further studies are also needed to explore if laser treatment is an adequate option in the maintenance phase after initial treatment.

Factors that might influence the results are that more smokers and also slightly more severe peri‐implantitis were found in the surgery group. These factors may have had a negative impact on the outcomes of especially the surgery group, since smoking has been recognized as an aggravating factor for developing both periodontitis and peri‐implantitis.^44^

A limitation in the present study, affecting the results generalizability, is the number of participants, where we planned to include 50 patients, but due to COVID‐19 pandemic, it was not possible. It has been shown that the pandemic has negatively impacted periodontal practices, especially in the public and academic sectors.^45^ On the other hand, Roccuzzo et al.^37^ investigated 25 participants in their study, and that numbers of participants seems to be in line with other RCTs in a single clinic setting in periodontology, although a few studies reach up to 60 participants.^6^ A 6‐month follow‐up period may be regarded as short, but is suggested as a minimum follow‐up time for evaluating treatment of peri‐implant diseases.^46^

## 5 CONCLUSIONS

The proportion of patients with improved clinical outcomes was similar between the 2 treatment groups, albeit surgical treatment yielded greater pocket reduction. No differences were found in immunological or microbial outcomes. Further studies are required to evaluate and optimize the laser protocol, assessing other wavelengths, as well as including more participants to minimize the risk of bias due to variations in plaque control, smoking, and other background characteristics.

## AUTHOR CONTRIBUTIONS

Sebastian Malmqvist – funding secured by, the planning of the study, performed baseline, first week of healing and 6 months follow‐up examinations, performed laser treatment and oral hygiene instructions, statistical analysis, data interpretation, writing of first draft of manuscript, and reviewing and editing manuscript text. Talat Qadri – funding secured by, the planning of the study, intervention calibration, data interpretation, and reviewing and editing manuscript text. Ronaldo Lira‐Junior – protocols for and performance of immunological analysis, assessing radiographs, data interpretation, and reviewing and editing manuscript text. Elisabeth A Boström – funding secured by, the planning of the study, the protocols for immunological analysis, data interpretation, and reviewing and editing manuscript text. Anders Gustafsson – funding secured by, the planning of the study, data interpretation, and reviewing and editing manuscript text. Georgios N Belibasakis – funding secured by, the planning of the study, protocols for microbiological sampling and analysis, data interpretation, and reviewing and editing manuscript text. Angelika Silbereisen—protocols for and performance of microbiological analysis, data interpretation, and reviewing and editing manuscript text. Gunnar Johannsen—funding secured by, the planning of the study, screening for potential participants, performed surgical intervention, data interpretation, and reviewing and editing manuscript text. Annsofi Johannsen—funding secured by, the planning of the study, assessing radiographs, data interpretation, and reviewing and editing manuscript text.

## CONFLICT OF INTEREST STATEMENT

The authors declare that they have no conflicts of interest.

## DATA AVAILABILITY STATEMENT

Data available on request from the authors.

## Supporting information



Supporting information

## Footnotes

* SiroLaser Blue, Dentsply Sirona, Bensheim, Germany.

† EasyTip 320, Dentsply Sirona, Bensheim, Germany.

‡ NiTi Brush Pocket, HANS KOREA Co. Ltd., Goyang, South Korea.

§ PCP‐UNC 15, Hu‐Friedy, Chicago, IL.

‖ Periopaper, Oraflow Incorporated, Smithtown, NY.

¶ Periotron 8000, Oraflow Incorporated, Smithtown, NY.

# R&D Systems, Minneapolis, MN.

** SpectraMAX 340, Sunnyvale, CA.

†† Top Dent Paper Points, Pearl Dent Co. LTD., Ho Chi Minh City, Vietnam.

‡‡ GenElute Bacterial Genomic DNA Kit NA2110, Sigma‐Aldrich, Merck KGaA, Darmstadt, Germany.

§§ PowerUp SYBR Green Master Mix, Thermo Fisher Scientific, Waltham, MA.

‖‖ IBM SPSS version 28.0, IBM Corp., Armonk, NY.
