# Supplementary material for: Treatment of peri‐implantitis with diode laser or mucosal flap surgery: A clinical randomized controlled trial
Source: J Periodontol. 2025 Mar 24;96(10):1126–37. doi: 10.1002/JPER.24-0683 (PMC12572678; doi:10.1002/JPER.24-0683)
Supplement: Supplementary file 1 — Supporting information [file JPER-96-1126-s001.docx]

# Supplementary Tables (1-5)

| **Supplementary Table 1. DNA standards and their respective primers.** | | | | | |
| --- | --- | --- | --- | --- | --- |
| **Bacteria** | **NCBI Taxonomy ID** | **Conc. [ng/μl]** | **Genome size [Mb]** | **Forward primer sequence** | **Reverse primer sequence** |
| *Porphyromonas gingivalis* | 431947 | 295.2 | 2.35 | 5'-CGATGATACGCGAGGAACCTTACCC | 5'-CCGAAGGGAAGAAGCTCTCATCT |
| *Fusobacterium nucleatum* | 76856 | 124.2 | 2.18 | 5'-CGCCCGTCACACCACGAGA | 5'-ACACCCTCGGAACACCCTCCTTAC |
| *Tannerella forsythia* | 203275 | 39.4 | 3.41 | 5’-GCGAGAGCCTGAACCAGCCA | 5’- ACTCGTATCGCCCGTTATTCCCGTA |
| *Treponema denticola* | 243275 | 9.4 | 2.84 | 5'-TAAGGGACAGCTTGCTCACCCCTA | 5'-CACCCACGCGTTACTCACCAGTC |

| **Supplementary Table 2. Full-mouth clinical and radiographic variables before and 6 months after treatment.** | | | | | | | | | |
| --- | --- | --- | --- | --- | --- | --- | --- | --- | --- |
| **Variable** | **Baseline (B)** | | | **6 months (6m)** | | | **Change 6m – B** | | |
|  | Laser | Surgery | p-value | Laser | Surgery | p-value | Laser | Surgery | p-value |
| PI [%] | 49.6 ± 28.6  (34.57, 64.56) | 49.7 ± 18.5  (39.22, 60.19) | 0.988 | 49.3 ± 20.8  (38.37, 60.15) | 46.4 ± 24.0  (32.78, 59.94) | 0.744 | -0.3 ± 24.6  (-13.21, 12.61)  p = 0.964 | -3.3 ± 19.0  (-14.11, 7.43)  p = 0.555 | 0.731 |
| BOP [%] | 47.1 ± 15.4  (39.0, 55.1) | 42.0 ± 15.5  (33.2, 50.8) | 0.415 | 46.2 ± 15.1  (38.3, 54.1) | 48.1 ± 22.9  (35.1, 61.0) | 0.804 | -0.9 ± 12.6  (-7.5, 5.7)  p = 0.796 | 6.0 ± 25.7  (-8.5, 20.6)  p = 0.850^†^ | 0.700^*^ |
| SOP [%] | 3.6 ± 4.3  (1.3, 5.8) | 3.0 ± 2.8  (1.4, 4.6) | 1.000^*^ | 3.9 ± 6.7  (0.4, 7.4) | 4.4 ± 5.4  (1.3, 7.5) | 0.432^*^ | 0.3 ± 3.1  (-1.4, 1.9)  **p = 0.009^†^** | 1.4 ± 3.5  (-0.5, 3.4)  **p = 0.004** | 0.336^*^ |
| PPD [mm] | 2.96 ± 0.69  (2.60, 3.32) | 2.85 ± 0.46  (2.59, 3.11) | 0.797^*^ | 2.98 ± 0.77  (2.57, 3.38) | 2.82 ± 0.53  (2.52, 3.12) | 0.738^*^ | 0.01 ± 0.17  (-0.08, 0.10)  p = 0.781 | -0.03 ± 0.24  (-0.17, 0.10)  p = 0.642 | 0.574 |
| 4-5mm PPD [n] | 10.7 ± 6.9  (7.1, 14.4) | 10.8 ± 8.7  (5.9, 15.8) | 0.969 | 10.9 ± 6.9  (7.3, 14.5) | 9.6 ± 6.9  (5.7, 13.5) | 0.625 | 0.2 ± 4.1  (-1.9, 2.4)  p = 0.847 | -1.3 ± 3.9  (-3.4, 0.9)  p = 0.437^†^ | 0.717^*^ |
| ≥ 6mm PPD [n] | 4.6 ± 3.8  (2.7, 6.6) | 6.0 ± 3.4  (4.1, 7.9) | 0.143^*^ | 4.0 ± 3.4  (2.3, 5.8) | 5.6 ± 4.3  (3.1, 8.0) | 0.304 | -0.6 ± 2.4  (-1.9, 0.6)  p = 0.342 | -0.4 ± 2.5  (-1.9, 1.0)  p = 0.581 | 0.819 |
| Values expressed as means ± standard deviation (95% CI). Independent t-test between groups and paired t-test baseline, 6 months. Significant *p*-values in bold.  ^*^Mann-Whitney U-test  ^†^Wilcoxon signed-rank test  Abbreviations: PI, plaque index; BOP, bleeding on probing; SOP, suppuration on probing; PPD, probing pocket depth. | | | | | | | | | |

| **Supplementary Table 3. Levels of biomarkers in saliva and PICF at baseline and after 6 months.** | | | | | | | | | |
| --- | --- | --- | --- | --- | --- | --- | --- | --- | --- |
| **Variable** | **Baseline (B)** | | | **6 months (6m)** | | | **Change (6m – B)** | | |
|  | Laser | Surgery | *p*1 | Laser | Surgery | *p*1 | Laser | Surgery | *p*1 |
| **Stim. saliva** |  |  |  |  |  |  |  |  |  |
| S100A8/A9  [ng/mL]  *p*2 | 11833.6 ± 9826.4  (6686.3, 16980.9) | 9444.6 ± 5585.1  (6284.6, 12604.6) | 0.781 | 9362.7 ± 6282.6  (6071.7, 12653.6) | 13901.3 ± 10874.2  (7748.7, 20053.8) | 0.274 | -2471.0 ± 7123.0  (-6202.2, 1260.3)  *p*=0.268 | 4456.7 ± 10399.3  (-1427.2, 10340.5)  *p*=0.176 | 0.085 |
| MMP-8  [ng/mL]  *p*2 | 83.1 ± 64.0  (49.6, 116.6) | 160.2 ± 259.8  (13.2, 307.2) | 0.432 | 86.9 ± 70.6  (50.0, 123.9) | 175.7 ± 217.2  (52.8, 298.6) | 0.374 | 3.8 ± 44.0  (-19.2, 26.9)  *p*=0.670 | 15.5 ± 219.0  (-108.4, 139.4)  *p*=0.910 | 0.860 |
| IL-1β  [pg/mL]  *p*2 | 70.0 ± 102.6  (16.2, 123.7) | 87.6 ± 96.5  (33.0, 142.1) | 0.328 | 92.1 ± 112.4  (33.3, 151.0) | 81.3 ± 66.5  (43.7, 118.9) | 0.571 | 22.1 ± 101.6  (-31.1, 75.4)  *p*=0.952 | -6.2 ± 108.9  (-67.9, 55.4)  *p*=0.339 | 0.667 |
| **PICF** |  |  |  |  |  |  |  |  |  |
| S100A8/A9  [ng/mL]  *p*2 | 9708.4 ± 5376.6  (6892.0, 12524.8) | 7759.8 ± 6291.5  (4200.1, 11319.4) | 0.643 | 12959.6 ± 5104.2  (10285.9, 15633.3) | 11003.9 ± 7314.4  (6865.4, 15142.4) | 0.820 | 3251.2 ± 5112.4  (573.2, 5929.2)  ***p*=0.035** | 3244.2 ± 7080.5  (-761.9, 7250.2)  *p*=0.176 | 1.000 |
| MMP-8  [ng/mL]  *p*2 | 102.2 ± 97.5  (51.2, 153.3) | 72.8 ± 109.3  (11.0, 134.7) | 0.252 | 105.4 ± 74.8  (66.2, 144.6) | 114.2 ± 102.3  (56.3, 172.1) | 0.959 | 3.2 ± 68.7  (-32.8, 39.1)  *p*=0.952 | 41.4 ± 118.1  (-25.4, 108.2)  *p*=0.266 | 0.297 |
| IL-1β  [pg/mL]  *p*2 | 224.4 ± 299.3  (67.6, 381.2) | 200.8 ± 237.5  (66.4, 335.1) | 0.781 | 183.6 ± 178.5  (90.2, 277.1) | 227.3 ± 231.2  (96.5, 358.1) | 0.860 | -40.7 ± 194.8  (-142.8, 61.3)  *p*=0.583 | 26.5 ± 262.9  (-122.2, 175.3)  *p*=0.791 | 0.595 |
| *Note:* Values expressed as mean concentration ± standard deviation (95% confidence interval). Mann-Whitney U-test *p*1 comparison between groups and Wilcoxon signed-rank test *p*2 change withing the groups from baseline to 6 months. Significant *p*-values in bold.  Abbreviations: Stim., stimulated; PICF, peri-implant crevicular fluid; S100A8/A9, calprotectin; IL, interleukin; MMP, matrix metalloproteinase. | | | | | | | | | |

| **Supplementary Table 4. Periotron values and volume of PICF at baseline and after 6 months.** | | | | | | | | | |
| --- | --- | --- | --- | --- | --- | --- | --- | --- | --- |
| **Variable** | **Baseline (B)** | | | **6 months (6m)** | | | **Change (6m – B)** | | |
|  | Laser | Surgery | *p*1 | Laser | Surgery | *p*1 | Laser | Surgery | *p*1 |
| Value [units]  *p*2 | 71.6 ± 41.3  (93.2, 50.0) | 74.7 ± 46.3  (48.5, 100.9) | 0.918 | 78.3 ± 54.7  (49.6, 106.9) | 88.1 ± 53.5  (57.9, 118.4) | 0.537 | 6.7 ± 40.9  (-14.7, 28.1)  *p*=0.950 | 13.4 ± 63.6  (-22.6, 49.4)  *p*=1.000 ^a^ | 0.817 |
| Volume [μl]  *p*2 | 0.53 ± 0.31  (0.37, 0.69) | 0.55 ± 0.34  (0.36, 0.75) | 0.918 | 0.58 ± 0.40  (0.37, 0.79) | 0.65 ± 0.40  (0.43, 0.88 | 0.537 | 0.05 ± 0.30  (-0.11, 0.21)  *p*=0.952 | 0.10 ± 0,47  (-0.17, 0.37)  *p*=1.000 | 0.938 |
| *Note:* Values expressed as means ± standard deviation (95% confidence interval). Mann-Whitney U-test *p*1 comparison between groups and Wilcoxon signed-rank test *p*2 change withing the groups from baseline to 6 months.  Abbreviations: PICF, peri-implant crevicular fluid | | | | | | | | | |

| **Supplementary Table 5. Counts (log10) of periodontal pathogens at baseline and after 6 months.** | | | | | | | | | |  |
| --- | --- | --- | --- | --- | --- | --- | --- | --- | --- | --- |
| **Variable** | **Baseline (B)** | | | **6 months (6m)** | | | **Change (6m – B)** | | | |
|  | Laser | Surgery | *p*1 | Laser | Surgery | *p*1 | Laser | Surgery | *p*1 | |
| P.g.  *p*2 | 3.38 ± 2.21  (2.18, 4.58) | 1.78 ± 1.78  (0.77, 2.79) | 0.052 | 3.15 ± 2.03  (2.05, 4.25) | 1.07 ± 1.54  (0.15, 1.98) | **0.030** | -0.23 ± 0.69  (-0.61, 0.15)  *p*=0.191 | -0.43 ± 0.46  (-0.71, -0.16)  *p*=0.123 | 0.252 | |
| F.n.  *p*2 | 4.68 ± 0.42  (4.45, 4.91) | 4.76 ± 0.58  (4.43, 5.09) | 0.936 | 4.65 ± 0.52  (4.36, 4.93) | 5.04 ± 1.21  (4.35, 5.72) | 0.574 | -0.03 ± 0.52  (-0.32, 0.25)  *p*=0.753 | 0.28 ± 1.22  (-0.41, 0.97)  *p*=0.622 | 0.936 | |
| T.f.  *p*2 | 4.17 ± 1.96  (3.11, 5.24) | 4.10 ± 1.74  (3.12, 5.09) | 0.470 | 4.65 ± 1.40  (3.85, 5.44) | 4.53 ± 1.22  (3.81, 5.25) | 0.651 | 0.10 ± 0.55  (-0.21, 0.41)  *p*=0.894 | 0.49 ± 0.95  (-0.07, 1.06)  *p*=0.102 | 0.235 | |
| T.d.  *p*2 | 4.70 ± 1.58  (3.84, 5.55) | 4.92 ± 1.50  (4.08, 5.77) | 0.611 | 4.69 ± 1.66  (3.79, 5.59) | 5.39 ± 0.73  (4.96, 5.82) | 0.228 | -0.01 ± 1.11  (-0.61, 0.59)  *p*=0.972 | 0.06 ± 0.80  (-0.41, 0.53)  *p*=0.638 | 1.000 | |
| *Note:* Values expressed as mean log_10_ counts ± standard deviation (95% confidence interval) in the pair-pooled submucosal biofilm samples from the dental implant with deepest radiographic bone defect. Mann-Whitney U-test *p*1 comparison between groups and Wilcoxon signed-rank test *p*2 change within the groups from baseline to 6 months. Significant *p*-values in bold.  Abbreviations: P.g., *Porphyromonas gingivalis*; F.n., *Fusobacterium nucleatum*; T.f., *Tannerella forsythia*; T.d., *Treponema denticola*. | | | | | | | | | | |
